# Ten years of invasion: *Harmonia axyridis* (Pallas) (Coleoptera: Coccinellidae) in Britain

**DOI:** 10.1111/een.12203

**Published:** 2015-05-20

**Authors:** Helen E Roy, Peter M J Brown

**Affiliations:** 1 Centre for Ecology & HydrologyOxfordshire, U.K.; 2 Animal and Environment Research Group, Life Sciences Department, Anglia Ruskin UniversityCambridge, U.K.

**Keywords:** Biological invasions, citizen science, intra-guild predation, invasive species, monitoring and surveillance, non-native species

## Abstract

1. *Harmonia axyridis* was first recorded in Britain in 2004. Two subsequent earlier records were received from 2003.

2. The UK Ladybird Survey, a citizen science initiative involving online recording, was launched in 2005 to encourage people across Britain to track the spread of *H. axyridis*. Tens of thousands of people have provided records of *H. axyridis* and other species of ladybirds, creating an invaluable dataset for large-scale and long-term research. Declines in the distribution of seven (of eight assessed) native species of ladybird have been demonstrated, and correlated with the arrival of *H. axyridis*, using the records collated through the UK Ladybird Survey.

3. Experimental research and field surveys have also contributed to our understanding of the ecology of *H. axyridis* and particularly the process of invasion. *Harmonia axyridis* arrived in Britain through dispersal and introduction events from regions in which it was deliberately released as a biological control agent. The rapid spread of this species has been attributed to its high natural dispersal capability by means of both flight and anthropogenic transport. A number of factors have contributed to the successful establishment and indeed dominance of this polymorphic species within aphidophagous guilds, including high reproductive capacity, intra-guild predation, eurytopic nature, high resistance to natural enemies within the invaded range, and potentially phenotypic plasticity.

4. The global invasion by *H. axyridis* and subsequent research on this species has contributed to the general understanding of biological invasions.

## Introduction

The first British record of *Harmonia axyridis* (Pallas) (Coleoptera: Coccinellidae), the harlequin ladybird, was from Sible Hedingham, Essex, England, in 2004 (Majerus *et al*., 2006). Widely introduced across continental Europe as a biological control agent of aphids, it had never been intentionally introduced into Britain (Brown *et al*., 2008a). However, it was perhaps inevitable that individuals from the introduced populations in Europe would arrive in Britain because *H. axyridis* has excellent dispersal abilities (Brown *et al*., 2008b; Jeffries *et al*., 2013).

The first record of *H. axyridis* within Britain, alongside the rapid spread of this species elsewhere in Europe, triggered a number of responses:

1. Rapid development of online recording and the launch of the UK Ladybird Survey including the Harlequin Ladybird Survey [encompassing the Biological Records Centre (BRC) hosted Coccinellidae Recording Scheme].
2. Research collaborations across Europe through the establishment of a working group ‘Risks and benefits of exotic biological control agents’ within the International Organisation for Biological and Integrated Control (IOBC).
3. Publication of a review in *Ecological Entomology* outlining potential impacts of the arrival of *H. axyridis* (Majerus *et al*., 2006).

The review published in *Ecological Entomology* (Majerus *et al*., 2006) outlined a number of predictions (Table 1) and provided a framework for research specifically within Britain but with relevance across Europe and beyond. Indeed, *H. axyridis* was noted as providing ‘entomologists with a unique and exciting opportunity to monitor the spread and impacts of an invasive alien insect in British environments that might prove a timely model study for future ecological impact assessments’. The long history of invasion of *H. axyridis* in America was highlighted in the review and it was recognised that there was much to be gained from comparative studies building on the research findings available from America (Koch & Galvan, 2008).

**Table 1:** Predictions following the arrival of *Harmonia axyridis* in Britain (Majerus *et al.*, 2006) alongside a summary of recent evidence, supporting references, and overall conclusions, based on current understanding, with respect to the importance of factors in determining success of invasion by this species

Prediction	Evidence	References	Conclusion
Eurytopic nature of *H. axyridis* will contribute to rapid spread	The range of host plant associations and widespread distribution of *H. axyridis* in Britain reflect the eurytopic nature of this species, although coniferous woodlands may negatively affect the spread of *H. axyridis*.	Brown *et al*. (2008b, 2011a)	+
	**Habitat breadth is an important factor contributing to the invasion success of *H. axyridis*.**		
Climatic adaptability of *H. axyridis* will give it a competitive advantage over some of the more niche-specific native ladybirds	Climatic conditions have not been a barrier to the colonisation and spread of *H. axyridis* in southern Britain, but are speculated to have limited its abundance in northern England and in Scotland.	Comont *et al*. (2012) and Purse *et al*. (2014)	+/?
	**There are clear discrepancies between the observed and predicted (climate model) distributions of *H. axyridis*, and it is apparent that climate is an important factor in determining the spread of this species but alongside other interacting biotic and abiotic factors.**		
Maritime climate of Britain will allow *H. axyridis* to breed throughout the summer, with no requirement for a summer dormancy	Continual breeding of this species is apparent and at least two generations of *H. axyridis* have been observed each year since arrival.	Brown *et al*. (2008b) and Roy *et al*. (2011a)	+
	**Multivoltinism contributes to the rapid rate of population growth of *H. axyridis* each year and, consequently, to spread.**		
Phenotypic plasticity will allow *H. axyridis* to successfully and regularly extend its breeding season to September, October, and even into November	Phenotypic plasticity displayed by *H. axyridis* enables local adaptation at temporal and spatial scales; increase in autumnal melanisation may have accelerated the spread of *H. axyridis*.	Michie *et al*. (2010) and Purse *et al*. (2014)	?
	**Further work is required to elucidate the importance of phenotypic plasticity in the invasion success of *H. axyridis*.**		
*H. axyridis* will spread across the entire British mainland by 2008	The first record of *H. axyridis* in Scotland was in 2007. However, there are relatively few records in Scotland and its distribution and breeding there are limited.	Brown *et al*. (2008a,2008b, 2011b) and Roy *et al*. (2011a)	+
	**High dispersal ability of this species has clearly been demonstrated in most of England and Wales.**		
Spread and increase of *H. axyridis* in Britain may therefore prove to be beneficial to crop systems by restricting aphid numbers below economically damaging levels and so reduce the use of chemical pesticides	Recent research highlights the importance of *H. axyridis* as an aphid predator in crop systems in the UK.	Wells (2011)	?
	**Further work is required to explore the ecosystem-level impact of *H. axyridis* on pest insects and particularly the ecosystem service provided by this alien predator.**		
*Harmonia axyridis* is likely to have a negative effect on other aphidophages in three ways: resource competition, intra-guild predation, and intraspecific competition	There is considerable evidence of intra-guild predation from laboratory and field observations.	Ware and Majerus (2008), Ware *et al*. (2009), Wells *et al*. (2010), Brown *et al*. (2011a), Wells (2011), Roy *et al*. (2012) and Brown *et al*. (2014)	+
	Observations from the UK Ladybird Survey highlight a strong correlation between the presence of *H. axyridis* and declines in the distribution of native ladybird species.		
	Further work is required on competitive interactions, although recent research in laboratory mesocosms suggests that high aphid density does not reduce intra-guild predation.		
	**There is considerable evidence of negative effects of *H. axyridis* on other species, but effects on ecosystem function require further work.**		
Efficient chemical defence and relatively large size would provide *H. axyridis* with a significant reproductive advantage over many native British species	A few studies indicate the importance of chemical defence and body size in intra-guild interactions.	Bezzerides *et al*. and (2007) and Ware *et al*. (2008)	+/?
	**The importance of chemical defence and large size in contributing to reproductive advantage of *H. axyridis* over native species requires further investigation.**		
*H. axyridis* will become a nuisance to humans	There have been many reports of *H. axyridis* forming large aggregations in domestic dwellings, and in some cases people have reported this species as a nuisance.	Roy *et al*. (2011a)	−
	**There is some evidence of negative effects on humans.**		

+, important factor; –, unimportant; ?, undecided.

*Harmonia axyridis* has been the inspiration and focus of research across the globe (Sloggett, 2005, 2012). Indeed, 19 papers were published in a special issue of the journal *BioControl* as a result of the collaboration through the IOBC working group ‘Risks and benefits of exotic biological control agents’ (Roy & Wajnberg, 2008). These publications have been widely cited and demonstrate the collaborative approach to research on *H. axyridis*. Here we provide an overview of research findings, particularly in Britain, over the last 10 years. We highlight the contributions made through research on *H. axyridis* to the field of invasion biology, focusing on predictions from Majerus *et al*. (2006); the manuscript has been structured to align with the review (Majerus *et al*., 2006).

## Factors affecting the population demography of *Harmonia axyridis* in Britain

**Fig. 1:**
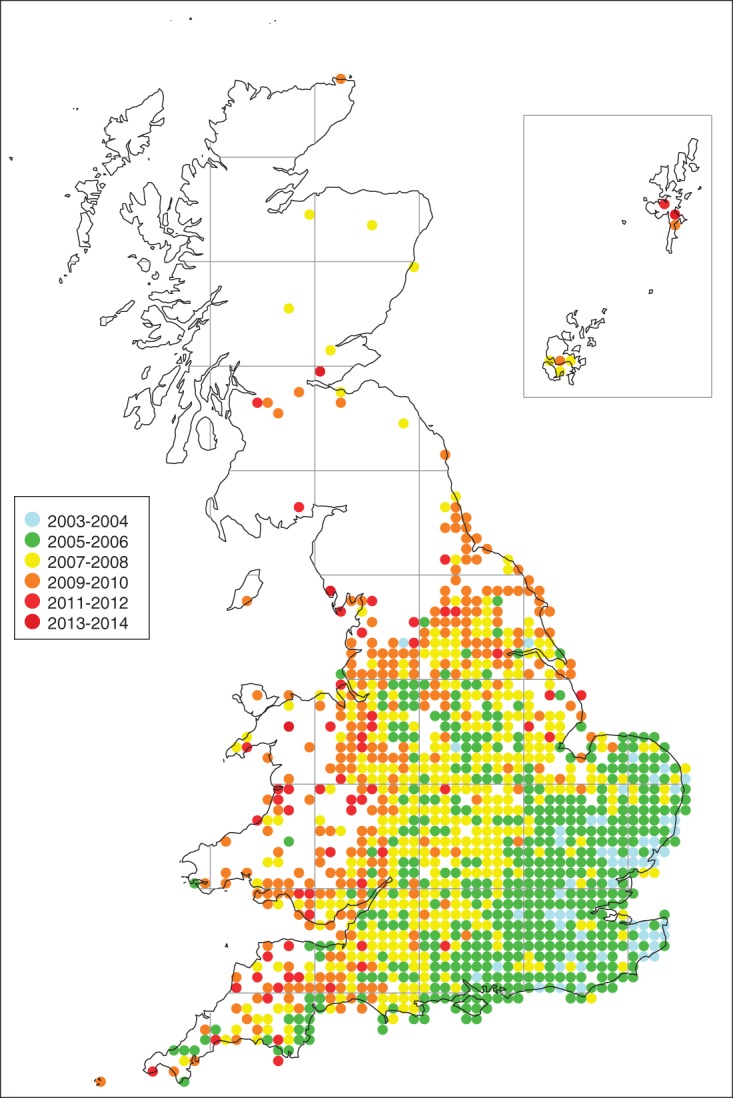
*Harmonia axyridis* occurrence in 10-km squares in Britain from 2004 to 2014. Where a square has been recorded in more than 1 year, occurrence in the earliest year is shown (blue, 2003–2004; green, 2005–2006; yellow, 2007–2008; orange, 2009–2010; red, 2011–2012; burgundy, 2013–2014).

The records of *H. axyridis* received through the UK Ladybird Survey have enabled the spread of this invader to be documented from early in the invasion process (Brown *et al*., 2008b). The considerable media attention in response to immediate notification of the arrival of *H. axyridis* in England led to approximately 100 verified records of the species from September to December 2004. These records were mainly from the south-east of England, with many from coastal areas, and only three outlying 10-km squares recorded in northern England (Brown *et al*., 2008b). *Harmonia axyridis* spread west and north within Britain, with the northerly spread rate from 2004 to 2008 calculated as 105 km year^–1^ (Brown *et al*., 2008b) and by 2009 was recorded in 1022 10-km squares encompassing all regions of England and Wales, with approximately 75% of 10-km squares within the invaded range having verified records (Fig. 1).

### Eurytopic nature

*Harmonia axyridis* is highly eurytopic in Britain, thriving in a wide range of habitats, as predicted by Majerus *et al*. (2006). It is particularly successful in urban localities, spreading faster into areas containing a high proportion of urban land (Purse *et al*., 2014). The species also thrives in rural locations; based on UK Ladybird Survey data, 19% of the 1-km squares with *H. axyridis* records were predominantly arable or horticultural land (Brown, 2010). The population dynamics of *H. axyridis* in crop systems (wheat, corn, broad bean, and potato crops) have been studied in Belgium and indicate that *H. axyridis* arrives 7–8 days after the dominant native coccinellids (Jansen & Hautier, 2008; Vandereycken *et al*., 2013). A 1-year study (2008) involving field observations in wheat and bean crops in southern England reported an absence of *H. axyridis* in wheat (aphid abundance was reported as low) but the presence of *H. axyridis* co-occurring with other coccinellids in bean crops (Wells, 2011). *Harmonia axyridis* was the most common aphidophagous species in bean crops, and the presence of this species was correlated with aphid abundance (Wells, 2011).

In Britain, 5% of *H. axyridis* records were from 1-km squares dominated by woodland (mostly broadleaved or mixed). The percentages of records submitted to the UK Ladybird Survey from various vegetation types were: deciduous trees and shrubs, 56%; herbaceous plants, 29%; evergreen trees and shrubs, 11%; grasses and others, 4% (Brown, 2010). Indeed, *H. axyridis* has been recorded from more than 75 plant families, dominated by Aceraceae (14% of records with associated plant data), Rosaceae (13%), and Malvaceae (10%) (Brown, 2010). Larvae of the species were recorded from about 50 of these families (Brown *et al*., 2011a), notably Aceraceae (22% of records with associated plant data), Malvaceae (18%), Rosaceae (10%) and Urticaceae, Betulaceae, and Salicaceae (5% each) (Brown, 2010). Thus, in Britain, *H. axyridis* thrives on deciduous trees and shrubs such as limes, maples, birches, and roses, as well as a variety of herbaceous plants, including stinging nettle. Similar patterns of host plant association have been observed in other regions of Europe (Roy *et al*., 2012; Panigaj *et al*., 2014). Records of *H. axyridis* from coniferous trees in Britain are quite limited, unlike in parts of its native range (Brown *et al*., 2011a).

The widespread distribution of *H. axyridis* in the UK reflects the eurytopic nature of this species. Indeed, the ability of *H. axyridis* to thrive in association with a diverse range of host plants undoubtedly explains the observed breadth of habitat types occupied by *H. axyridis*. A recent study on the spread of *H. axyridis*, including consideration of landscape factors, suggested that coniferous woodland, after correcting for bias in recording intensity, might negatively affect the spread of this species (Purse *et al*., 2014). Currently there is limited information within the UK Ladybird Survey database on specific plant associations, and what is there is mainly found in comment fields and so requires considerable work to extract (Brown, 2010). Further developments of the UK Ladybird Survey will include capturing information on plant associations as defined data fields within the online recording forms to enable future research on ecological networks.

### Climate

Climatic conditions have not been a barrier to the colonisation and spread of *H. axyridis* in southern Britain, but are speculated to have limited its abundance in northern England and in Scotland (Brown *et al*., 2008b). In these northern areas, records of successful breeding by *H. axyridis* are very limited. Climatic modelling studies have indicated that nearly all of mainland Britain is suitable for *H. axyridis*, with the exception of northern Scotland (Poutsma *et al*., 2008). The model proposed by Poutsma *et al*. (2008) has proved to be a good predictor of the expanding distribution of *H. axyridis* in Europe (Brown *et al*., 2011b), but the parameters used may need slight refinement to include observations on distribution from the UK Ladybird Survey. The combination of lower temperatures and higher precipitation in Scotland than in England appears to restrict *H. axyridis* to warm urban localities in Scotland, especially in terms of successful reproduction. The Orkney Islands and Shetland Islands (off the north-east coast of Scotland) are indicated as climatically unsuitable for *H. axyridis* (Poutsma *et al*., 2008); whilst there have been isolated records of individual adults from these northern islands, these ladybirds seem to have arrived on produce imported from the mainland (Ribbands *et al*., 2009) and there are no records here of juveniles of *H. axyridis*, or indeed of any other coccinellid species.

**Fig. 2:**
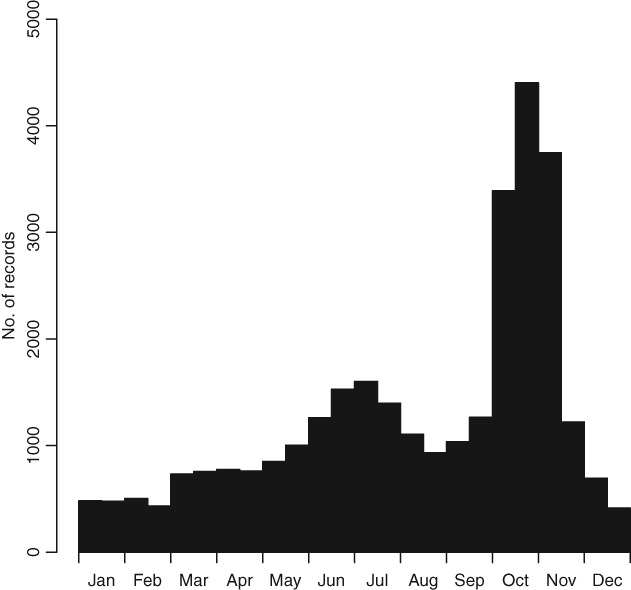
*Harmonia axyridis* phenogram displaying number of *H. axyridis* records in the UK Ladybird Survey database as monthly counts.

The most northerly record of *H. axyridis* in Europe is from Trondheim, Norway (Saethre *et al*., 2010), substantially further north than the Shetland Islands, but Oslo appears to be the most northerly location where *H. axyridis* has become established. Majerus *et al*. (2006) predicted that climate change may provide *H. axyridis* with a further competitive advantage over native British coccinellids. Predictions from modelling approaches suggest that *H. axyridis* may indeed benefit from climate warming through further northward expansion (Purse *et al*., 2014) and increased voltinism is also possible. As in parts of its native range, such as Japan (Osawa, 2011), *H. axyridis* is multivoltine in Britain and usually completes two generations per year (Brown *et al*., 2008b) (Fig. 2). Larval peaks are in June and October and there is the potential for three generations in particularly favourable years. Records of larvae in late December (winter) are not unusual. The resultant high population is presumed to encourage higher rates of dispersal for the species compared with native, univoltine species.

While climate models in part explain the distribution of *H. axyridis* within the invaded range, there are clear discrepancies between the observed and predicted distributions of *H. axyridis*. There are, of course, many factors that influence the invasion process and the distribution of species over time. Indeed, the interactions among landscape factors, climate, and species traits (such as polymorphism) in determining the distribution of ladybirds are complex (Comont *et al*., 2014a).

Summarising, the apparent lack of success of *H. axyridis* in Scotland could be attributed to a number of factors:

1. Biogeographic features such as mountain ranges may act as barriers to species dispersal and invasion (Wilson *et al*., 2009). Such barriers to *H. axyridis* in Britain include the Cambrian mountains (Wales) and particularly the Pennine mountains (northern England) (Brown *et al*., 2011b). The reasons why mountains may block dispersal are effectively encompassed within habitat and climatic limitations (see later).
2. Habitat factors such as soil type, land use, and vegetation type have a direct effect on ladybird prey species and therefore an indirect effect on ladybird populations (Comont *et al*., 2014a). Eurytopic ladybirds such as *H. axyridis* tend to thrive in habitats (such as arable and urban) with high prey abundance. In less favourable habitats such as those at higher altitudes (principally moorland and heathland in Britain), *H. axyridis* is found at lower densities, if at all.
3. The cooler and wetter climate typical of northern and upland regions of Britain is less favourable to many predatory insect species and their prey. Whilst the native range of *H. axyridis* includes southern Siberia, with very cold winter temperatures [e.g. mean January temperatures of –13 °C (daily high) to –18 °C (daily low) in Novosibirsk], it may be the combination of wet conditions with cold temperatures that is particularly unfavourable for *H. axyridis* in Scotland. Native ladybirds tend to be much lower in both species number and abundance in Scotland than in England; indeed, in Scotland, 25 of the 46 resident British coccinellid species are either absent or occur very rarely (Roy *et al*., 2011a).
4. Lower human population density in Scotland than in England and Wales could partially explain the low number of records in Scotland. This places a potential bias on our dataset, as clearly we would expect to receive fewer records from less populated areas. However, there are many 10-km squares in Scotland with high numbers of records of other species of ladybird, suggesting that the level of recording intensity is sufficient to derive a robust assessment of the distribution of *H. axyridis* throughout Britain.

There is still much to reveal about the spread of *H. axyridis* and it is important to recognise that invasions are dynamic processes. The UK Ladybird Survey dataset has been used in many studies exploring the interactions between abiotic and biotic factors in determining the distribution of ladybirds (Brown *et al*., 2008b; Brown, 2010; Purse *et al*., 2014; Comont *et al*., 2014a) and demonstrates the huge value of such citizen science initiatives for continued analysis of large-scale and long-term ecological processes.

### Phenotypic adaptability

There have been some intriguing insights into the phenotypic adaptability of *H. axyridis* over the past 10 years. Perhaps the most compelling evidence of phenotypic adaptability is in relation to colour pattern polymorphism. *Harmonia axyridis* is a polymorphic species for both the pattern and colour of the pronota and elytra (Majerus *et al*., 2006). Three main colour morphs have been reported in Britain: f. *succinea*, f. *spectabilis*, and f. *conspicua*. Additionally, there are a few records of f. *equicolor* and f. *aulica*; the nominate form – f. *axyridis* – has not been reported in Britain. The non-melanic f. *succinea* is the most abundant colour form and comprises approximately 80% of records (Brown *et al*., 2008b; Purse *et al*., 2014). The influence of temperature on f. *succinea* is intriguing; individual *H. axyridis* f. *succinea* (non-melanic) eclosing from pupae late in the year have larger spots than those eclosing in spring and early summer (Michie *et al*., 2010). Recent research has indicated that the phenotypic plasticity displayed by *H. axyridis* enables local adaptation at temporal and spatial scales (Michie *et al*., 2010), whereby melanism, which may be important in thermoregulation (Brakefield & de Jong, 2011), is considered costly in summer and beneficial in winter (Michie *et al*., 2010). Michie *et al*. (2010) proposed that melanisation may have accelerated the spread of *H. axyridis*.

Modelling approaches using the UK Ladybird Survey dataset have explored the prediction that melanic colour forms have a thermal regulatory advantage and consequently spread more rapidly than the non-melanic colour form (Purse *et al*., 2014). It was apparent that while increased sunshine significantly enhanced the spread of the non-melanic form (f. *succinea*), the spread was more rapid within hectads containing a high proportion of urban land cover and marginally slower in hectads containing high conifer cover (Purse *et al*., 2014). Additional recent research suggests that the colour pattern polymorphism of *H. axyridis* and variation in other life-history traits could contribute to the invasion success of this species (Majerus *et al*., 2006; Michie *et al*., 2010; Purse *et al*., 2014).

There have been a number of studies exploring the influence of life-history traits on the distribution of coccinellids including *H. axyridis* (Comont *et al*., 2012, 2014a). The traits database compiled for these studies provides a rare opportunity to explore variation in life-history traits between species. Indeed, it has been insightful to include information on species traits alongside the distribution data from the UK Ladybird Survey to explain trends in the distribution patterns of ladybirds in Britain (Comont *et al*., 2014a). Climate and habitat datasets available for Britain have added further value to these analyses (Comont *et al*., 2014a). It would be fascinating to extend this research beyond Britain to consider life-history traits in a biogeographic context. Additionally, modelling approaches enable eloquent exploration of large-scale and long-term datasets to test predictions and ultimately construct further hypotheses. Detailed empirical approaches are required to examine the mechanisms at play.

### Dispersal potential

The rapid spread of *H. axyridis* has been a consequence of both natural dispersal by flight and anthropogenic processes. Recent research using innovative research tools, namely vertical-looking entomological radar, have provided intriguing insights into the flight patterns of *H. axyridis*. *Harmonia axyridis* and *C. septempunctata* were detected at 1100 m above ground level moving at 60 km h^−1^ and sustaining flight for up to 2 h, indicating a high capacity for long-distance dispersal.

Much of Britain is densely populated and has an elaborate transport network, and there are many reports of *H. axyridis* being transported accidentally in or on vehicles. Inadvertent movement with people and goods has undoubtedly facilitated the spread of the species in Britain. For example, in 2007, *H. axyridis* was first recorded from Scotland (Holroyd *et al*., 2008) as a result of the ladybird being transported in a suitcase; the first record from the Orkney islands (northern Scotland, 2008; Ribbands *et al*., 2009) and from Northern Ireland (2007; Murchie *et al*., 2008) involved the ladybirds being transported with vegetables from mainland Britain. The most noticeable record in this regard is that of the population that was initiated by transport of ladybirds to a supermarket in Derby (north-central England) in 2004. The beetles spread rapidly from there and are likely to have accelerated the northerly spread of the species from 2005.

### Natural enemy interactions

Natural enemy escape provides an appealing hypothesis for explaining the success of an invader (Roy *et al*., 2011b). The Enemy Release Hypothesis (ERH) (Elton, 1958; Torchin *et al*., 2003) predicts that an alien species will be less affected by specialised natural enemies (predators, parasites and pathogens) than will native species. Thus, the alien gains a competitive advantage and may rapidly increase in abundance and distribution (Elton, 1958; Torchin *et al*., 2003; Colautti *et al*., 2004). The premise of this theory is that natural enemies are important in regulating populations (Roy & Lawson Handley, 2012), but the empirical evidence for this, and consequently the ERH, is limited (Roy & Cottrell, 2008; Roy *et al*., 2011b). There have been some advances in understanding the role of natural enemies in the *H. axyridis* invasion over the last 10 years (Kenis *et al*., 2008; Roy *et al*., 2013; Comont *et al*., 2014b).

Arguably the most important natural enemies of ladybirds in Britain are pathogens (such as *Beauveria bassiana*) and several species of endoparasitic Hymenoptera and Diptera (Roy *et al*., 2011a, 2013; Ceryngier *et al*., 2012; Comont *et al*., 2014b). There were early indications that some of the ladybird's natural enemies native to Britain would attack *H. axyridis* (Hall *et al*., 2009; Ware *et al*., 2010), but laboratory studies indicated the low susceptibility of this invader in comparison to native species (Koyama & Majerus, 2008; Roy *et al*., 2008b). Laboratory research indicates that *H. axyridis* is an unfavourable host for *Dinocampus coccinellae* (Schrank) (Hymenoptera: Braconidae) (Hoogendoorn & Heimpel, 2002; Berkvens *et al*., 2010b). The exact mechanism involved in the resistance of *H. axyridis* to *D. coccinellae* is unclear, but teratocyte cells produced by *D. coccinellae* (involved in both immunosuppression of the host and nutrition of the parasitoid) follow an abnormal pattern of growth within *H. axyridis*, which could explain the impeded development of *D. coccinellae* within this invader (Firlej, 2012). The fungal pathogen *B. bassiana* commonly infects native species of ladybird (such as *C. septempunctata*), but again *H. axyridis* appears to be highly resistant (Roy *et al*., 2008b). A recent study also demonstrated that *H. axyridis* individuals contain high numbers of obligate parasitic microsporidia, which appear to have no adverse affects on *H. axyridis* but cause high mortality when artificially injected into *C. septempunctata* (Vilcinskas *et al*., 2013). The ecological relevance of this study requires further investigation because injection is an artificial process and far removed from the natural mechanisms involved in microsporidia transmission.

Clearly there is a need to extend studies of natural enemies to the field in order to ensure ecological relevance. One recent study from Britain confirmed low rates of parasitism of *H. axyridis*, particularly in comparison to the native *C. septempunctata* (Comont *et al*., 2014b). Indeed, pupae of *H. axyridis* were parasitised, primarily by *Phalacrotophora fasciata* (Fallén) and *Phalacrotophora berolinensis* Schmitz (Diptera: Phoridae), at an exceptionally low level (1.73%) and adults were not found to be parasitised at all in this study. In contrast, parasitism of the co-occurring *C. septempunctata* was high (20.91% pupae, 5.67% adults). This provides evidence in support of the ERH, i.e. success of the invader may result from a reduction or absence of natural enemies (Elton, 1958; Torchin *et al*., 2001). However, further work is required to elucidate population-level effects of this difference in parasitism rates between the alien and native species. There is no doubt that *H. axyridis* represents an excellent opportunity to explore natural enemy interactions and their role in the invasion process.

## Impacts

### Benefits as a pest control agent

There has been little focus in Britain on the role of *H. axyridis* as a beneficial pest control agent (Wells, 2011). The effects of *H. axyridis* on aphid populations in British crop systems are unknown and are worthy of further investigation, particularly with respect to ecosystem services and resilience (Koch & Galvan, 2008; Vilà *et al*., 2009).

### Negative effects on pest and non-pest herbivorous insects

*Harmonia axyridis* has a wide diet breadth (reviewed by Hodek & Evans, 2012) and, in the absence of aphids, can complete development on a combination of other foods including coccids, adelgids, psyllids and many other insects, including conspecifics (Tedders & Schaefer, 1994; Koch, 2003; Flowers *et al*., 2005; Onofre Soares *et al*., 2005; Majerus *et al*., 2006; Hodek & Evans, 2012), but also *Ephestia kuehniella* (Lepidoptera: Pyralidae) eggs (Berkvens *et al*., 2010a) and pollen (Berkvens *et al*., 2008, 2010a). Therefore, *H. axyridis* is predicted to pose a threat to many species. However, there have been few studies exploring the population-level effects of *H. axyridis* on non-target herbivorous insects. One study recognised the potential for *H. axyridis* to negatively affect monarch butterflies, *Danaus plexippus* (L.) (Lepidoptera: Nymphalidae), in the US (Koch *et al*., 2003). In the UK Ladybird Survey database, there are records of *H. axyridis* predating lepidopterans (such as the eggs of noctuid moths) in Britain, but the extent of such predation is unknown. Further research is required to examine the population dynamics of these interacting species. It is also important to note that *H. axyridis* is not unique amongst the coccinellids in having a wide diet breadth (Sloggett, 2012).

### Negative effects on other aphidophages

*Harmonia axyridis* is widely recognised as a top predator within aphidophagous guilds (Pell *et al*., 2008). However, as highlighted by Majerus *et al*. (2006), the negative effects of *H. axyridis* are likely to be the result of a complex range of interactions, with *H. axyridis* having a competitive edge through resource competition, intra-guild predation (IGP), and a more plastic phenotype than other aphidophagous species. There have been many published studies exploring such interactions, particularly IGP, and to a lesser extent, competition (Phoofolo & Obrycki, 1998; Ware *et al*., 2009). Initially these were mostly small-scale laboratory studies of ladybird interactions within Petri dishes, which demonstrated strong asymmetric IGP in favour of *H. axyridis* over native ladybirds (Pell *et al*., 2008; Ware & Majerus, 2008; Ware *et al*., 2008; Roy *et al*., 2008a). In contrast, it is apparent that in such Petri dish experiments *Chrysoperla carnea* (Stephens) (Neuroptera: Chrysopidae) is an intra-guild predator of *H. axyridis* (Nedvěd *et al*., 2013), but in mesocosm experiments IGP was in favour of *H. axyridis* over *C. carnea* (Wells, 2011).

Understanding of intra-guild interactions has progressed by increasing the scale with the use of more realistic experimental arenas than Petri dishes. Mesocosm studies have included interactions between coccinellids and non-coccinellid aphidophages such as neuropterans (Wells *et al*., 2010; Wells, 2011) and syrphids (Ingels & De Clercq, 2011). Such approaches to exploring IGP are critical for informing risk assessment by enabling rapid assessment of interactions for a range of potential prey species and different life stages of both the intra-guild predator and prey. Additionally, assessing the effects of aphid density on IGP provides further context to the experiments (Ware *et al*., 2009; Wells, 2011), but results so far suggest that the prevalence of IGP is not reduced by increased aphid density (Wells, 2011). However, extrapolating findings from laboratory studies to the field is challenging and many questions remain with respect to the ecological relevance of IGP. Molecular tools provide exciting opportunities for investigating community interactions in the field (Roy & Lawson Handley, 2012). Analyses using the polymerase chain reaction have been employed to detect prey DNA from the guts of field-collected *H. axyridis* samples. Initial work in Britain assessed larval gut contents for two intra-guild prey – *Adalia bipunctata* (L.) (Coleoptera: Coccinellidae) and *Adalia decempunctata* (L.) (Coleoptera: Coccinellidae) – and both were detected within *H. axyridis* (Thomas *et al*., 2013). This work was extended to investigate predation of neuropterans and syrphids by *H. axyridis* with testing of samples from five European countries (Brown *et al*., 2014). Through this study it was apparent that while syrphids were detected in the gut of *H. axyridis*, neuropterans were not. Gas chromatography-mass spectrometry has been used in mainland Europe for the detection of ladybird IGP and revealed similar results (Hautier *et al*., 2011).

The taxonomic breadth of studies demonstrating IGP by *H. axyridis* supports the contention that *H. axyridis* is an aggressive coccinellid with a tendency for intra-guild predation that could seriously affect the abundance of native coccinellids and dramatically reduce their available niches in the predator complex (Elliott *et al*., 1996). Furthermore, observations from the UK Ladybird Survey (Roy *et al*., 2012) highlight the potential for *H. axyridis* to dramatically disrupt native guilds in Britain (Majerus *et al*., 2006). However, further understanding of the implications of IGP by *H. axyridis* on ecological resilience and function should be prioritised. Recent research from America found no evidence that *H. axyridis* consumed coccinellid eggs in the field, but suggested that exploitative and apparent competition might explain declines of native species in the presence of *H. axyridis* (Smith & Gardiner, 2013). There is an urgent need for detailed field studies to quantitatively document the interactions between invaders and other species within the community. Ecological network analysis provides exciting opportunities for detailed exploration of the complex interactions across the aphidophagous community (Roy & Lawson Handley, 2012). It will be particularly intriguing to explore the concept of ecological resilience and extend research on ecological networks to consideration of other invaded systems (Romanuk *et al*., 2009).

### Negative effects on humans

Overwintering aggregations of *H. axyridis* have undoubtedly been one of the most notable aspects of invasion by this species. Many people report sightings from their houses during autumn and winter to the UK Ladybird Survey, with the annual peak of records generally being in late October and early November. Many people have reported problems associated with overwintering aggregations of *H. axyridis*, specifically staining of soft furnishings and unpleasant smell associated with the secretion of reflex blood. There have been observations of thousands of individuals inside houses and in the bell towers and porches of churches (Roy *et al*., 2011a). However, the impacts on people, beyond a minor nuisance, are limited in the British context. Allergic reactions to *H. axyridis* are rare (Goetz, 2008) but there have been a few reports of such reactions in Britain.

In wine-growing regions of North America, *H. axyridis* has attained the status of a pest (Koch *et al*., 2004; Koch & Galvan, 2008). This is not the case for all vineyard owners, some of whom have looked on its appearance in their (British) vineyards favourably (DeCourcy, 2009), despite concerns elsewhere over negative effects on wine production (Galvan *et al*., 2008). There are no known reports of negative impacts in vineyards in Britain, where grape-growing is rare. *Harmonia axyridis* has a tendency to aggregate on soft fruits, including grapes, and exacerbates damage through feeding, but also contaminates the crop because it is difficult to separate the beetles at harvest. The tainting caused by *H. axyridis* crushed with the grapes is problematic. However, recently concerns have been raised that it is not just *H. axyridis* that causes such problems in North America, but also *C. septempunctata*, native to Britain but an alien species in North America (Botezatu *et al*., 2013). Both *H. axyridis* and *C. septempunctata* contribute alkyl methoxypyrazines, and particularly isopropyl methoxypyrazine, to wine at concentrations that are considered to have a negative impact on wine quality (Botezatu *et al*., 2013). Although there are no effective and recommended control strategies available for *H. axyridis* (Kenis *et al*., 2008), there are indications that sulphur dioxide (in the form of potassium metabisulphite), a commonly used antimicrobial and antioxidant in wine production, repels *H. axyridis* from grapevines (Glemser *et al*., 2012).

### Potential control strategies

Methods for controlling the spread of *H. axyridis* have been proposed (Kenis *et al*., 2008). *Harmonia axyridis* produces an aggregation pheromone to attract other individuals to overwinterwing habitats (Verheggen *et al*., 2007). The use of the aggegration pheromone within a network of traps has been proposed and could potentially work at a local scale (such as in a vineyard, where preventing *H. axyridis* from aggregating within bunches of grapes would be advantageous). However, at a large scale there would be practical implications that would render this approach unfeasible; a very large number of traps would be needed and the costs involved in managing the traps would be prohibitively high.

There are a number of natural enemies of *H. axyridis* that could potentially exert control, but population-level effects of natural enemies on the regulation of ladybirds are poorly understood (Roy *et al*., 2011c; Comont *et al*., 2014b). Additionally, as outlined earlier, studies on the interactions between *H*. *axyridis* and natural enemies strongly indicate that *H. axyridis* is less susceptible to attack by native parasitoids and pathogens than are native ladybirds, although this may change in the future. The ectoparasitic mite *Coccipolipus hippodamiae* (McDaniel & Morrill) (Acarina: Podapolipidae) naturally occurs in Europe and causes sterility in female *H. axyridis* (Rhule *et al*., 2010). Therefore, this mite has been considered as a biological control candidate (Rhule *et al*., 2010). However, some native ladybird species are also susceptible to it. Whilst *H. axyridis* may be more susceptible because of the nature of its life cycle, rigorous risk assessments would be needed before any artificial releases of the mite are considered (Rhule *et al*., 2010), and in our opinion the mite represents a control strategy that is too risky.

## Implications for invasion biology

*Harmonia axyridis* was speculated as a model species for understanding invasion (Roy & Wajnberg, 2008). The unified framework for invasion biology proposed by Blackburn *et al*. (2011) recognises that the invasion process can be considered as discrete stages and that there are barriers a species must overcome to establish and subsequently spread. There are many ways in which research on invasion by *H. axyridis* has provided evidence to underpin mechanisms of invasion (Table 2). From the transport of this invader beyond the limits of its native geographic range to the dramatic spread of this species within the invaded range, there has been extensive ecological research documenting the processes and exploring the underlying mechanisms of invasion. However, there are still many knowledge gaps and opportunities for studies on *H. axyridis* contributing to our understanding of invasion biology.

**Table 2:** Examples of studies on *Harmonia axyridis* over the last 10 years that have provided evidence to underpin understanding of the invasion process (Blackburn *et al.*, 2011)

Stage of invasion	Barrier	Evidence
Transport	Geography	The Altai mountains provide a biogeographic barrier to spread from the native range, but introduction as a biological control agent enabled the global spread of *H. axyridis* (Brown *et al*., 2011b).
	*Species that has been transported beyond the limits of its native geographic range and that has established a population in an area where it was not known to occur previously*.	Accidental transport from continental Europe to Britain alongside natural dispersal contributed to the arrival of *H. axyridis* in 2004 (Brown *et al*., 2008b).
Introduction	Captivation or cultivation	Introductions of *H. axyridis* in continental Europe were predominantly in glasshouses for the control of aphids, but individuals could have escaped into the wider countryside (Adachi-Hagimori *et al*., 2011).
	*Species can be prevented from becoming an invader by a human-imposed barrier. Many animal and plant species exist in captivity and/or cultivation beyond the limits of their native ranges, but fail to cross the physical barriers of a fence or hedge. This barrier is probably lower for species in cultivation than for those in captivity*	Many widespread invasions arise from successful invasive populations rather than directly from the native range (invasive bridgehead effect) and this has been demonstrated for *H. axyridis*. An invasive population in eastern North America appears to have been the source that invaded the European, South American, and African continents, with some admixture with a biological control strain in Europe (Lombaert *et al*., 2010).
Establishment	Survival	Successful overwintering in Britain since 2004–2005 (Brown *et al*., 2008b)
	*Introduced population can fail to establish because individuals in the population fail to survive. Failure to establish can result from factors associated with the species (e.g. reproductive rate or specialism), the location (e.g. presence of enemies or mutualists), apparently stochastic features of the individual introduction event (especially propagule pressure) or, often, their interaction (e.g. species location, such as climate matching); these factors can act on survival or reproduction, or both*.	*Harmonia axyridis* is climatically matched with most regions of the world, including mainland Britain (Poutsma *et al*., 2008).
		Low susceptibility to natural enemies within the invaded range (Roy *et al*., 2008b, 2011b,2011c; Berkvens *et al*., 2010b; Comont *et al*., 2014b).
	Reproduction	Successful breeding in Britain since 2005 (Brown *et al*., 2008b)
	*An introduced population can fail to establish because individuals in the population either fail to survive, or survive but fail to reproduce*.	Multivoltine species (Brown *et al*., 2008b)
Spread	Dispersal	Ability to exploit resources in a wide range of habitats has ensured spread across Britain but limited spread north of Pennine and west of Cambrian mountains (Brown *et al*., 2011b).
	*A spreading population essentially faces multiple, sequential establishment events, under an ever greater range of environmental conditions*.	Low susceptibility to natural enemies within the invaded range (Koyama & Majerus, 2008; Roy *et al*., 2008b; Berkvens *et al*., 2010b; Comont *et al*., 2014b).
		*Harmonia axyridis* are able to travel 18 km in a ‘typical’ high-altitude flight, but up to 120 km if flying at higher altitudes, indicating a high capacity for long-distance dispersal (Jeffries *et al*., 2013).
	Environmental	Exploitation of buildings as favourable overwintering location (Brown *et al.,* 2008a)
	*The invasive range is determined by the extent of suitable environment, and the environmental barrier sets the limits to this*.	Exploitation of wide range of habitats, especially anthropogenic ones, including urban and crop systems (Brown *et al*., 2011b).

The stage of invasion and barrier (with extracts of the relevant text provided in italics) are defined by Blackburn *et al*. (2011), and selected evidence derived from research on *H. axyridis* is outlined.

## Future directions: the next 10 years

The arrival of *H. axyridis* in Britain was met with trepidation; indeed, in the press release announcing the arrival of *H. axyridis*, Professor Michael Majerus described this species as ‘the most invasive ladybird on Earth’. The dramatic spread of *H. axyridis* suggests that it is one of the fastest-spreading invaders worldwide and is worthy of this description. However, *H. axyridis* has successfully been used as a model invasive alien species and has been the inspiration for global collaborations; the last decade of research is indicative of the enthusiasm and commitment of many biologists. Nevertheless, there is scope to expand the collaborations, particularly to increase the breadth of parallel studies conducted in the native and invaded regions. A recent symposium on *H. axyridis* in China (International Congress on Biological Invasions, Qingdao, 23–27 October 2013) highlighted the willingness for such global collaboration and the insights that can be gained from scientists working across Asia.

There have been an impressive number of studies on *H. axyridis* over the last 10 years that have provided mechanistic evidence (Table 1) alongside models explaining large-scale patterns and processes. The potential of IGP as an important force structuring aphidophagous communities has been highlighted, but understanding of the ecological relevance of IGP across complex networks of species is lacking. Additionally, the relative importance of competition and IGP should be assessed; indeed, it is thought that competitive interactions might be more important than IGP in driving declines of native species (Smith & Gardiner, 2013). The numerical dominance of *H. axyridis* in many habitats across Britain is evident, but the effects of the species on ecosystem function are unclear. There are clear indications that *H. axyridis* is escaping natural enemies within the invaded range but over the next 10 years it would seem plausible that the natural enemies will begin to adapt. Indeed, *H. axyridis* represents an abundant resource for parasites and pathogens.

*Harmonia axyridis* has provided unique and detailed insights into invasion biology over the decades, and the demand for scientific evidence to underpin invasion biology will undoubtedly be high over the next 10 years. In recent years, the European Commission (EC) has intensified its commitment to providing a comprehensive and manageable solution to invasive alien species in Europe. A European Union (EU) Regulation (http://ec.europa.eu/environment/nature/invasivealien/index_en.htm) has recently been adopted. Scientifically robust risk assessments, as laid down in the Regulation, will be essential. The number of records of *H. axyridis* received by the UK Ladybird Survey demonstrates the critical role that people can play in alien species surveillance. Such surveillance is critical to strategies for early-warning and rapid response. A recent horizon-scanning exercise has highlighted the species most likely to arrive, establish, and threaten biodiversity within the next 10 years, and the top 30 species include six terrestrial invertebrates (Roy *et al*., 2014).

## References

[b1] Adachi-Hagimori T, Shibao M, Tanaka H, Seko T, Miura K (2011). Control of *Myzus persicae* and *Lipaphis erysimi* (Hemiptera: Aphididae) by adults and larvae of a flightless strain of *Harmonia axyridis* (Coleoptera: Coccinellidae) on non-heading *Brassica* cultivars in the greenhouse. BioControl.

[b2] Berkvens N, Bonte J, Berkvens D, Deforce K, Tirry L, De Clercq P (2008). Pollen as an alternative food for *Harmonia axyridis*. From Biological Control to Invasion: The Ladybird Harmonia Axyridis as a Model Species.

[b3] Berkvens N, Landuyt C, Deforce K, Berkvens D, Tirry L, De Clercq P (2010a). Alternative foods for the multicoloured Asian lady beetle *Harmonia axyridis* (Coleoptera: Coccinellidae). European Journal of Entomology.

[b4] Berkvens N, Moensa J, Berkvens D, Samihc MA, Tirry L, De Clercq P (2010b). *Dinocampus coccinellae* as a parasitoid of the invasive ladybird *Harmonia axyridis* in Europe. Biological Control.

[b5] Bezzerides AL, McGraw KJ, Parker RS, Husseini J (2007). Elytra color as a signal of chemical defense in the Asian ladybird beetle *Harmonia axyridis*. Behavioral Ecology and Sociobiology.

[b6] Blackburn TM, Pyšek P, Bacher S, Carlton JT, Duncan RP, Jarošík V (2011). A proposed unified framework for biological invasions. Trends in Ecology & Evolution.

[b7] Botezatu AI, Kotseridis Y, Inglis D, Pickering GJ (2013). Occurrence and contribution of alkyl methoxypyrazines in wine tainted by *Harmonia axyridis* and *Coccinella septempunctata*. Journal of the Science of Food and Agriculture.

[b8] Brakefield PM, De Jong PW (2011). A steep cline in ladybird melanism has decayed over 25 years: a genetic response to climate change?. Heredity.

[b9] Brown PMJ (2010).

[b10] Brown PMJ, Adriaens T, Bathon H, Cuppen J, Goldarazena A, Hagg T (2008a). *Harmonia axyridis* in Europe: spread and distribution of a non-native coccinellid. BioControl.

[b11] Brown PMJ, Roy HE, Rothery P, Roy DB, Ware RL, Majerus MEN (2008b). *Harmonia axyridis* in Great Britain: analysis of the spread and distribution of a non-native coccinellid. BioControl.

[b12] Brown PMJ, Frost R, Doberski J, Sparks T, Harrington R, Roy HE (2011a). Decline in native ladybirds in response to the arrival of *Harmonia axyridis*: early evidence from England. Ecological Entomology.

[b13] Brown PMJ, Thomas CE, Lombaert E, Jeffries DL, Estoup A, Handley L-JL (2011b). The global spread of *Harmonia axyridis* (Coleoptera: Coccinellidae): distribution, dispersal and routes of invasion. BioControl.

[b14] Brown PMJ, Ingels B, Wheatley A, Rhule EL, De Clercq P, van Leeuwen T (2014). Intraguild predation by Harmonia axyridis (Coleoptera: Coccinellidae) on native insects in Europe: molecular detection from field samples. Entomological Science.

[b15] Ceryngier P, Roy HE, Poland RL (2012). Natural enemies of ladybird beetles.

[b16] Colautti RI, Ricciardi A, Grigorovich IA, Macisaac HJ (2004). Is invasion success explained by the enemy release hypothesis?. Ecology Letters.

[b17] Comont RF, Roy HE, Lewis OT, Harrington R, Shortall CR, Purse BV (2012). Using biological traits to explain ladybird distribution patterns. Journal of Biogeography.

[b18] Comont R, Roy H, Harrington R, Shortall C, Purse B (2014a). Ecological correlates of local extinction and colonisation in the British ladybird beetles (Coleoptera: Coccinellidae). Biological Invasions.

[b19] Comont RF, Purse BV, Phillips W, Kunin WE, Hanson M, Lewis OT (2014b). Escape from parasitism by the invasive alien ladybird, Harmonia axyridis. Insect Conservation and Diversity.

[b20] Decourcy G (2009).

[b21] Elliott N, Kieckhefer R, Kauffman W (1996). Effects of an invading coccinellid on native coccinellids in an agricultural landscape. Oecologia.

[b22] Elton CS (1958). The Ecology of Invasions by Animals and Plants.

[b23] Firlej I (2012). Teratocytes growth pattern reflects host suitability in a host–parasitoid assemblage. Annals of the Entomological Society of America.

[b24] Flowers R, Salom S, Kok L (2005). Competitive interactions among two specialist predators and a generalist predator of hemlock woolly adelgid, *Adelges tsugae* (Homoptera: Adelgidae), in the laboratory. Environmental Entomology.

[b25] Galvan TL, Koch RL, Hutchison WD (2008). Impact of fruit feeding on overwintering survival of the multicolored Asian lady beetle, and the ability of this insect and paper wasps to injure wine grape berries. Entomologia Experimentalis et Applicata.

[b26] Glemser EJ, Dowling L, Inglis D, Pickering GJ, McFadden-Smith W, Sears MK (2012). A novel method for controlling multicolored Asian lady beetle (Coleoptera: Coccinellidae) in vineyards. Environmental Entomology.

[b27] Goetz DW (2008). *Harmonia axyridis* ladybug invasion and allergy. Allergy and Asthma Proceedings.

[b28] Hall R, Ware R, Michie LJ (2009). First record of field parasitism of immature stages of the Harlequin Ladybird *Harmonia axyridis* (Pallas) (Col.: Coccinellidae) by the braconid wasp *Dinocampus coccinellae* (Shrank) (Hym.: Braconidae). Entomologist's Record and Journal of Variation.

[b29] Hautier L, San Martin G, Callier P, De Biseau J-C, Grégoire J-C (2011). Alkaloids provide evidence of intraguild predation on native coccinellids by *Harmonia axyridis* in the field. Biological Invasions.

[b30] Hodek I, Hodek I, van Emden HF, Honek A, Evans EW (2012). Food relationships. Ecology and Behaviour of the Ladybird Beetles (Coccinellidae).

[b31] Holroyd O, Brown PMJ, Roy HE, Majerus MEN (2008). The harlequin ladybird, *Harmonia axyridis*, reaches Scotland. Entomologist's Record and Journal of Variation.

[b32] Hoogendoorn A, Heimpel G (2002). Indirect interactions between an introduced and a native ladybird beetle species mediated by a shared parasitoid. Biological Control.

[b33] Ingels B, De Clercq P (2011). Effect of size, extraguild prey and habitat complexity on intraguild interactions: a case study with the invasive ladybird Harmonia axyridis and the hoverfly Episyrphus balteatus. BioControl.

[b34] Jansen JP, Hautier L (2008). Ladybird population dynamics in potato: comparison of native species with an invasive species, *Harmonia axyridis*. BioControl.

[b35] Jeffries DL, Chapman J, Roy HE, Humphries S, Harrington R, Brown PMJ (2013). Characteristics and drivers of high-altitude ladybird flight: insights from vertical-looking entomological radar. PLoS ONE.

[b36] Kenis M, Roy HE, Zindel R, Majerus MEN (2008). Current and potential management strategies against *Harmonia axyridis*. BioControl.

[b37] Koch RL (2003). The multicolored Asian lady beetle, *Harmonia axyridis*: a review of its biology, uses in biological control, and non-target impacts. Journal of Insect Science.

[b38] Koch RL, Galvan TL (2008). Bad side of a good beetle: the North American experience with *Harmonia axyridis*. BioControl.

[b39] Koch RL, Hutchison WD, Venette RC, Heimpel GE (2003). Susceptibility of immature monarch butterfly, *Danaus plexippus* (Lepidoptera: Nymphalidae: Danainae), to predation by *Harmonia axyridis* (Coleoptera: Coccinellidae). Biological Control.

[b40] Koch R, Burkness E, Burkness SJW, Hutchison W (2004). Phytophagous preferences of the multicolored Asian lady beetle (Coleoptera: Coccinellidae) for autumn-ripening fruit. Journal of Economic Entomology.

[b41] Koyama S, Majerus MEN (2008). Interactions between the parasitoid wasp *Dinocampus coccinellae* and two species of coccinellid from Japan and Britain. BioControl.

[b42] Lombaert E, Guillemaud T, Cornuet J-M, Malausa T, Facon B, Estoup A (2010). Bridgehead effect in the worldwide invasion of the biocontrol harlequin ladybird. PLoS ONE.

[b43] Majerus M, Strawson V, Roy H (2006). The potential impacts of the arrival of the harlequin ladybird, *Harmonia axyridis* (Pallas) (Coleoptera: Coccinellidae), in Britain. Ecological Entomology.

[b44] Michie LJ, Mallard F, Majerus MEN, Jiggins FM (2010). Melanic through nature or nurture: genetic polymorphism and phenotypic plasticity in *Harmonia axyridis*. Journal of Evolutionary Biology.

[b45] Murchie AK, Moore JP, Moore GA, Roy HE (2008). The harlequin ladybird (*Harmonia axyridis* (Pallas)) (Coleoptera: Coccinellidae), found in Ireland. Irish Naturalists' Journal.

[b46] Nedvěd O, Xenia F, Ungerova D, Kalushkov P (2013). Alien vs. Predator–the native lacewing *Chrysoperla carnea* is the superior intraguild predator in trials against the invasive ladybird *Harmonia axyridis*. Bulletin of Insectology.

[b47] Onofre Soares A, Coderre D, Schanderl H (2005). Influence of prey quality on the fitness of two phenotypes of *Harmonia axyridis* adults. Entomologia Experimentalis et Applicata.

[b48] Osawa N (2011). Ecology of *Harmonia axyridis* in natural habitats within its native range. BioControl.

[b49] Panigaj L, Zach P, Honěk A, Nedvěd O, Kulfan J, Martinková Z (2014). The invasion history, distribution and colour pattern forms of the harlequin ladybird beetle *Harmonia axyridis* (Pall.)(Coleoptera, Coccinellidae) in Slovakia, Central Europe. ZooKeys.

[b50] Pell JK, Baverstock J, Roy HE, Ware RL, Majerus MEN (2008). Intraguild predation involving *Harmonia axyridis*: a review of current knowledge and future perspectives. BioControl.

[b51] Phoofolo MW, Obrycki JJ (1998). Potential for intraguild predation and competition among predatory Coccinellidae and Chrysopidae. Entomologia Experimentalis et Applicata.

[b52] Poutsma J, Loomans AJM, Aukema B, Heijerman T (2008). Predicting the potential geographical distribution of the harlequin ladybird, *Harmonia axyridis*, using the CLIMEX model. BioControl.

[b53] Purse BV, Comont R, Butler A, Brown PMJ, Kessel C, Roy HE (2014). Landscape and climate determine patterns of spread for all colour morphs of the alien ladybird Harmonia axyridis. Journal of Biogeography.

[b54] Rhule EL, Majerus ME, Jiggins FM, Ware RL (2010). Potential role of the sexually transmitted mite *Coccipolipus hippodamiae* in controlling populations of the invasive ladybird *Harmonia axyridis*. Biological Control.

[b55] Ribbands B, Brown PMJ, Roy HE, Majerus MEN (2009). The most northerly record of the Harlequin ladybird (Col., Coccinellidae) in the British Isles. Entomologist's Monthly Magazine.

[b56] Romanuk TN, Zhou Y, Brose U, Berlow EL, Williams RJ, Martinez ND (2009). Predicting invasion success in complex ecological networks. Philosophical Transactions of the Royal Society of London, Series B: Biological Sciences.

[b57] Roy HE, Cottrell TE (2008). Forgotten natural enemies: interactions between coccinellids and insect-parasitic fungi. European Journal of Entomology.

[b58] Roy HE, Lawson Handley L-J (2012). Networking: a community approach to invaders and their parasites. Functional Ecology.

[b59] Roy HE, Wajnberg E (2008). From biological control to invasion: the ladybird *Harmonia axyridis* as a model species. BioControl.

[b60] Roy HE, Baverstock J, Ware RL, Clark SJ, Majerus MEN, Baverstock KE (2008a). Intraguild predation of the aphid pathogenic fungus *Pandora neoaphidis* by the invasive coccinellid *Harmonia axyridis*. Ecological Entomology.

[b61] Roy HE, Brown PMJ, Rothery P, Ware RL, Majerus MEN (2008b). Interactions between the fungal pathogen *Beauveria bassiana* and three species of coccinellid: *Harmonia axyridis**Coccinella septempunctata* and *Adalia bipunctata*. BioControl.

[b63] Roy HE, Brown PMJ, Frost R, Poland RL (2011a). Atlas of the Ladybirds (Coccinellidae) of Britain and Ireland.

[b64] Roy HE, Handley LJL, Schoenrogge K, Poland RL, Purse BV (2011b). Can the enemy release hypothesis explain the success of invasive alien predators and parasitoids?. BioControl.

[b65] Roy HE, Rhule E, Harding S, Handley L-JL, Poland RL, Riddick EW (2011c). Living with the enemy: parasites and pathogens of the ladybird Harmonia axyridis. BioControl.

[b66] Roy HE, Adriaens T, Isaac NJB, Kenis M, Onkelinx T, San Martin G (2012). Invasive alien predator causes rapid declines of native European ladybirds. Diversity and Distributions.

[b67] Roy HE, Brown PMJ, Comont RF, Poland RL, Sloggett JJ, Majerus M (2013). Naturalists' Handbook 10: Ladybirds.

[b68] Roy HE, Peyton J, Aldridge DC, Bantock T, Blackburn TM, Britton R (2014). Horizon scanning for invasive alien species with the potential to threaten biodiversity in Great Britain. Global Change Biology.

[b69] Saethre MG, Staverløkk A, Hofsvang T (2010). The history of *Harmonia axyridis* (Pallas 1773) in Norway. IOBC/WPRS Bulletin.

[b70] Sloggett JJ (2005). Are we studying too few taxa? Insights from aphidophagous ladybird beetles (Coleoptera: Coccinellidae). European Journal of Entomology.

[b71] Sloggett JJ (2012). Harmonia axyridis invasions: deducing evolutionary causes and consequences. Entomological Science.

[b72] Smith CA, Gardiner MM (2013). Biodiversity loss following the introduction of exotic competitors: does intraguild predation explain the decline of native lady beetles?. PLoS ONE.

[b73] Tedders WL, Schaefer PW (1994). Release and establishment of *Harmonia axyridis* (Coleoptera, Coccinellidae) in the southeastern United States. Entomological News.

[b74] Thomas AP, Trotman J, Wheatley A, Aebi A, Zindel R, Brown PMJ (2013). Predation of native coccinellids by the invasive alien *Harmonia axyridis* (Coleoptera: Coccinellidae): detection in Britain by PCR-based gut analysis. Insect Conservation and Diversity.

[b75] Torchin M, Lafferty K, Kuris A (2001). Release from parasites as natural enemies: increased performance of a globally introduced marine crab. Biological Invasions.

[b76] Torchin ME, Lafferty KD, Dobson AP, McKenzie VJ, Kuris AM (2003). Introduced species and their missing parasites. Nature.

[b77] Vandereycken A, Brostaux Y, Joie E, Haubruge E, Verheggen FJ (2013). Occurrence of *Harmonia axyridis* (Coleoptera: Coccinellidae) in field crops. European Journal of Entomology.

[b78] Verheggen FJ, Fagel Q, Heuskin S, Lognay G, Francis F, Haubruge E (2007). Electrophysiological and behavioral responses of the multicolored Asian lady beetle, Harmonia axyridis Pallas, to sesquiterpene semiochemicals. Journal of Chemical Ecology.

[b79] Vilà M, Basnou C, Pyšek P, Josefsson M, Genovesi P, Gollasch S (2009). How well do we understand the impacts of alien species on ecosystem services? A pan-European, cross-taxa assessment. Frontiers in Ecology and the Environment.

[b80] Vilcinskas A, Stoecker K, Schmidtberg H, Rohrich CR, Vogel H (2013). Invasive harlequin ladybird carries biological weapons against native competitors. Science.

[b81] Ware RL, Majerus MEN (2008). Intraguild predation of immature stages of British and Japanese coccinellids by the invasive ladybird *Harmonia axyridis*. BioControl.

[b82] Ware RL, Ramon-Portugal F, Magro A, Ducamp C, Hemptinne JL, Majerus MEN (2008). Chemical protection of *Calvia quatuordecimguttata* eggs against intraguild predation by the invasive ladybird *Harmonia axyridis*. BioControl.

[b83] Ware RL, Yguel B, Majerus MEN (2009). Effects of competition, cannibalism and intra-guild predation on larval development of the European coccinellid *Adalia bipunctata* and the invasive species *Harmonia axyridis*. Ecological Entomology.

[b84] Ware R, Michie L, Otani T, Rhule E, Hall RJ (2010). Adaptation of native parasitoids to a novel host: the invasive coccinellid *Harmonia axyridis*. IOBC Bulletin.

[b85] Wells PM (2011).

[b86] Wells PM, Baverstock J, Majerus MEN, Jiggins FM, Roy HE, Pell JK (2010). Intraguild predation of non-coccinellid aphid natural enemies by *Harmonia axyridis*: prey range and factors influencing intraguild predation. IOBC/WPRS Bulletin.

[b87] Wilson JRU, Dormontt EE, Prentis PJ, Lowe AJ, Richardson DM (2009). Something in the way you move: dispersal pathways affect invasion success. Trends in Ecology & Evolution.

